# Biomass Carbon Magnetic Adsorbent Constructed by One-Step Activation Method for the Removal of Hg^0^ in Flue Gas

**DOI:** 10.1021/acsomega.1c05857

**Published:** 2022-03-10

**Authors:** Yu Cui, Qihuang Huo, Huijun Chen, Shuai Chen, Sheng Wang, Jiancheng Wang, Liping Chang, Lina Han, Wei Xie

**Affiliations:** † College of Materials Science and Engineering, Taiyuan University of Technology, Taiyuan 030024, China; ‡ State Key Laboratory of Clean and Efficient Coal Utilization, Taiyuan University of Technology, Taiyuan 030024, China; § Analytical Instrumentation Center, Institute of Coal Chemistry, Chinese Academy of Sciences, Taiyuan 030032, China; ∥ Dalian National Laboratory for Clean Energy, Dalian Institute of Chemical Physics, Chinese Academy of science, Dalian 116023, China; ⊥ Chemical Engineering, University of Newcastle, Callaghan NSW 2308, Australia

## Abstract

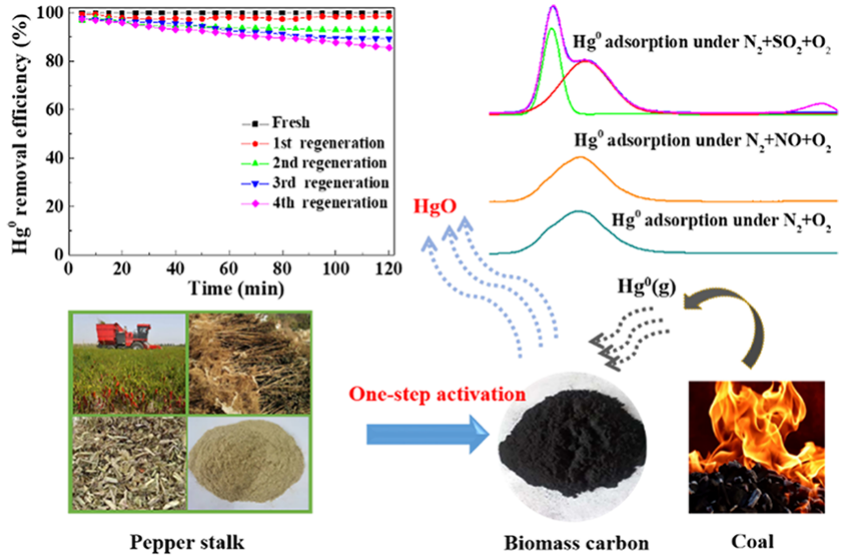

Elemental mercury (Hg^0^) emission from industrial boilers equipped in factories such as coal-fired power plants poses serious hazards to the environment and human health. Herein, an iron-modified biomass carbon (Fe/BC) magnetic adsorbent was prepared by a one-step method using pepper straw waste as raw material and potassium oxalate and ferric nitrate as activator and catalyst precursor, respectively. A fixed-bed reactor was used to evaluate the Hg^0^ removal performance of the Fe/BC adsorbent. The synthesized adsorbent showed a wide temperature window for Hg^0^ removal. In a N_2_ + O_2_ atmosphere, the removal efficiency toward Hg^0^ was 97.6% at 150 °C. Further, O_2_ or SO_2_ could promote the removal of Hg^0^, while NO could inhibit the conversion of Hg^0^ over the Fe/BC adsorbent. The consequence of XPS and Hg-TPD showed that lattice oxygen in Fe_2_O_3_ and chemisorbed oxygen were the main active sites for Hg^0^ removal, and HgO was the main mercury species on used Fe/BC. Moreover, Fe/BC adsorbent showed a good regeneration and magnetization performance, which was conducive to the cost reduction of actual industrial application. This study provides a facile approach for efficient removal of Hg^0^ using biomass-derived carbon material.

## 1 Introduction

Mercury (Hg), an important lethal pollutant, is a trace heavy metal element and displays toxicity, for instance, persistence, mobility, and bioaccumulation in both food chains and ecosystems.^1,2^ The anthropogenic Hg emissions basically originate from coal combustion. The proportion of it in total emissions is around 30%.^3^ There are three forms of mercury in flue gas: mercury oxide (Hg^2+^), particle bound mercury (Hg^p^), and elemental mercury (Hg^0^).^4−6^ It is well-known that Hg^p^ and Hg^2+^ can be easily controlled by dust removal devices and wet flue gas desulfurization (WFGD), respectively.^7,8^ Nevertheless, Hg^0^ is difficult to remove because it is insoluble and volatile at normal temperature and pressure.^9,10^ Consequently, development of systems that ensure the removal of Hg^0^ is imperative.

In recent years, for the removal efficiency of Hg^0^, substantial related technologies from adsorption^11^ to catalytic oxidation^12^ have been applied. Adsorption capture is considered as one of the most promising ones in all technologies. For the removal of Hg^0^ in coal combustion flue gas, the adsorbents have been accessed by many investigators, for example, activated carbons (AC),^11,13^ mineral materials,^14^ zeolites,^15^ calcium sorbents,^16^ petroleum coke,^17^ and fly ash.^18^ Among these, AC has been regarded as one of the most effective materials for the removal of Hg^0^. However, the high operating cost of conventional AC limits its large-scale applications in power plants. Therefore, discussions regarding the development of low-cost adsorbents have become a dominant research area in recent years.

As a renewable resource, biomass has been widely applied in the preparation of carbon-based adsorbents for catalysis and adsorption reactions because of its abundance, sustainability, economic benefits, and environmentally benign nature.^19^ According to statistics, China’s pepper planting area ranks second among vegetables. Because of its high carbon content and low ash content, this area has the potential to prepare biomass carbon.^20^ Pepper straw also contains different phenolic, carboxyl, ether, and amine groups, which may adsorb toxic elements in the environment.^20^ Therefore, the use of pepper straw for biomass carbon production is a feasible approach in terms of industrial waste management and renewable material development for Hg^0^ elimination from flue gas.

Raw biomass carbon shows a low Hg^0^ adsorption capacity because of its poor surface active sites. The surface active sites of biomass carbon can be improved by chemical means so as to effectively improve the adsorption performance of Hg^0^. Chemical activation can introduce active groups on the biomass carbon via acid,^21−23^ alkali,^24,25^ metal,^26−28^ sulfur,^29−31^ and halogen^32−34^ modifications, which promotes the removal of Hg^0^. Although the biomass adsorbents can be used as viable substitutes for AC, the powdered biomass carbon injected into flue gas will be captured by dust control devices together with fly ash, which is not conducive to the reuse of fly ash. Recently, to more efficiently recycle the used adsorbent from fly ash, some cheap magnetic mercury sorbents have been developed.^35−37^ For example, Yang et al.^35^ combined magnet cobalt iron impregnated porous carbon, and the active components of Hg^0^ capture were chemisorbed oxygen and lattice oxygen derived from Co_3_O_4_, Fe_3_O_4_, and Fe_2_O_3_. Xu et al.^38^ claimed that the preparation of a magnet organic-based carbon adsorbent by one-step pyrolysis of organic matter containing FeCl_3_ showed that Fe_3_O_4_ and or Cl^–^ could accurately improve the removal rate of Hg^0^. Shan et al.^39^ compounded a magnetic Mn–Fe biomass-based carbon adsorbent for capturing Hg^0^ from flu gas, and chemisorbed oxygen, lattice oxygen and active species on adsorbent surface were profitable for Hg^0^ removal. This research further inspired us to explore new methods of preparing magnetic biomass-based adsorbents through a simple preparation process that offers low cost and good regeneration performance to meet practical industrial applications. Ferrous nitrate Fe(NO_3_)_3_ is a safe and cheap chemical reagent that is commonly used as an oxidant in organic synthesis and a precursor of Fe_3_O_4_/γ-Fe_2_O_3_.^40^

On the basis of the above-mentioned discussions, it can be concluded that iron-modified biomass carbon (Fe/BC) is a type of good magnetic adsorbent for Hg^0^ removal. Agricultural waste is an important carbon source of biomass carbon. Pepper is widely planted all over the world, and pepper straws constitute a widespread agricultural waste. Therefore, in this study, Fe/BC magnetic adsorbent derived from pepper straw was prepared by a one-step method using K_2_C_2_O_4_ and Fe(NO_3_)_3_ as the activator and catalyst precursor, respectively. Biomass carbon was used for the removal of Hg^0^ in flue gas. In a specific reactor, the impact of flue gas composition (SO_2_, NO) and adsorption temperature on Hg^0^ removal efficiency at temperatures from 60 to 180 °C can be studied. On the basis of X-ray diffraction (XRD), N_2_ adsorption desorption, vibrating sample magnetometry (VSM), scanning electron microscopy (SEM), mercury temperature-programmed desorption (TPD), and X-ray photoelectron spectroscopy (XPS) techniques, the mechanism of Hg^0^ capture by Fe/BC adsorbent was deduced.

## 2 Experimental Section

### 2.1 Materials

Ferric nitrate nonahydrate (Fe(NO_3_)_3_·9H_2_O, AR) and potassium oxalate monohydrate (K_2_C_2_O_4_·H_2_O, AR) were purchased from Tianjin Kemiou Chemical Reagent Co. Raw materials (pepper straw) from a farm in Henan Province, China, were dried at 80 °C for 24 h and then crushed and sifted to a size of 0.25–0.42 mm. The proximate and ultimate analysis of the pepper straw was reported in Table 1. The ash content of pepper straw was up to 10.41 wt %. The content of volatile matter was high, which was beneficial for the pore formation of biomass carbon.^41^ The contents of C and O in pepper straw were high, while the contents of N and S were very low.

**Table 1:** Proximate and Ultimate Analyses of Pepper Straw

Proximate analysis (wt %, ad^a^)			Ultimate analysis (wt %, daf^a^)				
M	A	V	C	H	O^b^	N	S
4.89	10.41	66.47	47.01	5.9	44.78	1.92	0.39

a ad: air-dried basis. daf: dry and ash-free basis.

b Calculated by difference.

### 2.2 Preparation of Iron-Modified Biomass Carbon

Fe/BC adsorbents were prepared via a chemical activation method. K_2_C_2_O_4_ was selected as an activator. K_2_C_2_O_4_ decomposed into K_2_CO_3_ at low temperature, and the generated K_2_CO_3_ further corroded carbon. Further, as a pore-forming agent, the released CO is devoted to development of the porous structure.^42^ During the impregnation process of activator K_2_C_2_O_4_, Fe(NO_3_)_3_ was simultaneously added to prepare Fe/BC. This one-step activation and modification could reduce the times of calcination. The specific steps are as follows: First, pepper straw (3 g) was thoroughly mixed with an appropriate amount of K_2_C_2_O_4_ solution and Fe(NO_3_)_3_ solution, and then the mixed material solution was allowed to sit at room temperature for 10 h. Second, based on a certain temperature procedure and N_2_ atmosphere, the mixture was treated in a tube furnace; the mixture was heated to 30 °C directly and maintained at that temperature for 2 h before being cooled. The treated sample was ultimately washed with deionized until the pH was close to 7. The sample was dried at 80 °C for 12 h. The prepared Fe/BC adsorbent was named A_*x*_-Fe_*y*_-Tz, where *x* and *y* represent the concentrations of K_2_C_2_O_4_ and Fe(NO_3_)_3_ solutions, respectively; *T* represents the activation temperature; and *z* represents the specific temperature. For example, A_0.075_-Fe_0.4_-T850 adsorbent indicates that pepper straw was activated at 850 °C and K_2_C_2_O_4_/Fe(NO_3_)_3_ = 0.075:0.4 (concentration ratio).

### 2.3 Elemental Mercury Removal Experiments of Iron-Modified Biomass Carbon Adsorbents

The tests for Hg^0^ removal of Fe/BC adsorbents can be accessed in a fixed-bed system at laboratory scale. The evaluation apparatus reported in our previous study was used herein.^43^ In the test steps, the total simulated flue gas flow was controlled at 1000 mL min^–1^. The flue gas flow was 4 vol % O_2_, 0.02 vol % SO_2_ (when used), and 0.04 vol % NO (when used), 40 ± 2 μg m^–3^ Hg^0^ vapor, and N_2_. Through the precision of the mass flow controller, the flow rate of each gas was easily controlled. The Fe/BC adsorbent (0.16 g, 0.8 mL) was injected into a quartz reactor with a diameter of 8.0 mm. Then the simulated flue gas was brought into the reactor at an estimated temperature.

At the inside and outside of this reactor, the Hg^0^ concentrations were used in the Hg analyzer. Because of the removal performance of Hg^0^, the adsorbents’ activity was measured (η). The performance could be calculated following eq 1:
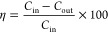
1In the flue gas, η is the removal performance of Hg^0^, *C*_in_ is the inside concentration, and *C*_out_ is the outside concentrations (μg m^–3^) of Hg^0^.

### 2.4 Characterization

By using the Micromeritics ASAP-2460 analyzer to pass the N_2_ adsorption–desorption test at −196 °C, the pore characteristics of the Fe/BC adsorbent could be decided. Using a diffractometer with curved graphite monochromatic Cu Kα radiation (λ = 0.15406 nm), the crystallinity and dispersion of the adsorbent were measured under the conditions of 40 kV and 15 mA, and XRD (Miniflex 600, Rigaku, Japan) spectra were obtained. The scan rate was 10°/min, and the range was 2θ (5–85°). The surface elemental properties of O, Fe, and Hg on samples were analyzed by XPS (ESCALAB 250Xi, Thermo Scientific, USA) with Al Kα radiation. The 284.8 eV C 1s peak calibration combined energy could be used. The magnetic properties of the Fe/BC adsorbent were determined using a VSM (lakeshore 735). SEM (JSM-7900F, JPN) was helpful for analyzing and describing the morphology characteristic of the adsorbents.

## 3 Results and Discussion

### 3.1 Elemental Mercury Capture

#### 3.1.1 Effect of Preparation Conditions on Elemental Mercury Removal

**Figure 1:**
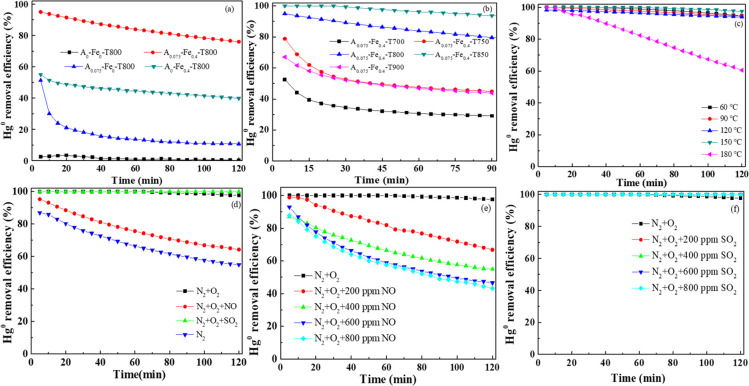
Hg^0^ removal efficiency of adsorbents using different activators (a); adsorbents with different activation temperature (b); adsorption temperature (c); atmosphere (d); NO concentration (e); and SO_2_ concentration (f). Experimental conditions: Hg^0^ inlet concentration 40 ± 2 μg m^–3^, 600 mL min^–1^ carrier N_2_, balance N_2_, 4 vol % O_2_, (a) *T* = 150 °C, (b) *T* = 150 °C, (c) *T* = 60–180 °C, (d) *T* = 150 °C, 200 ppm of SO_2_ (when used), 400 ppm of NO (when used), (e) *T* = 150 °C, 200–800 ppm of NO (when used), (f) *T* = 150 °C, 200–800 ppm of SO_2_ (when used)

The removal performance of Hg^0^ of Fe/BC magnetic adsorbents, which were prepared at different doses of activator K_2_C_2_O_4_ and catalyst precursor Fe(NO_3_)_3_, was assessed in a N_2_–O_2_–Hg atmosphere at 150 °C. Figure 1a shows the results, and the sample A_0_-Fe_0_-T800 (without K_2_C_2_O_4_ and Fe(NO_3_)_3_) exhibited a rather low Hg^0^ removal performance of about 0.4% in 2 h. The removal efficiency of adsorbent for Hg^0^ was improved at different levels after chemical activation. The Hg^0^ removal efficiency of sample A_0.075_-Fe_0_-T800 decreased from 51.3% to 10.1% within 2 h. These results showed that the removal activity of the Hg^0^ of the adsorbent prepared with the addition of K_2_C_2_O_4_ activator was better than that of the adsorbent without the addition of any activator in 2 h. The removal performance of Hg^0^ of the sample A_0_-Fe_0.4_-T800 decreased from 55.0% to 40.1%, indicating that the removal efficiency of Hg^0^ of the adsorbent prepared using only Fe(NO_3_)_3_ was better than that using only K_2_C_2_O_4_ as activator. The Hg^0^ removal efficiency of sample A_0.075_-Fe_0.4_-T800 was maintained at above 75.9% within 2 h. The results showed that the adsorbent prepared by adding K_2_C_2_O_4_ and Fe(NO_3_)_3_ exhibited the best Hg^0^ removal activity; the reason may be that K_2_C_2_O_4_ contributes to the pore development, and Fe(NO_3_)_3_ was decomposed into Fe_2_O_3_ during activation process, which had good mercury removal ability.^44^

Figure 1b shows the effect of activation temperature (700, 750, 800, 850, and 900 °C) on the removal efficiency of Hg^0^ of Fe/BC. It can be seen that the removal performance of Hg^0^ of sample A_0.075_-Fe_0.4_-T700 was lower than other samples. As the activation temperature increased from 750 to 800 and 850 °C, the removal efficiency of Hg^0^ increased from 44.1% to 75.9% and 97.6%, respectively. The experimental results showed that the high activation temperature had an apparent impact on the removal of Hg^0^. However, when the activation temperature gradually increased from 850 to 900 °C, the removal efficiency of Hg^0^ dropped from 97.6% to 44.2%. This result indicated that the sample carbonized at 850 °C exhibited the highest removal efficiency of Hg^0^. Thus, A_0.075_-Fe_0.4_-T850 adsorbents were used for subsequent experiments.

#### 3.1.2 Effect of Adsorption Temperature on Elemental Mercury Removal

Hg^0^ removal experiments were conducted at five different temperatures to analyze the effect of adsorption temperature on Hg^0^ removal efficiency, i.e., 60, 90, 120, 150, and 180 °C. Figure 1c shows the results. It can be seen that the removal efficiency of Hg^0^ of Fe/BC adsorbent increased with an increase in temperature from 60 to 150 °C. At 150 °C, the removal efficiency of Hg^0^ was the highest (97.6%). Xie et al.^45^ claimed that a higher temperature was beneficial for accelerating the chemical reaction rate between Hg^0^ and the active sites on the adsorbent. Nevertheless, the removal performance of Hg^0^ of A_0.075_-Fe_0.4_-T850 adsorbent was lower at 180 °C than that at 150 °C. The performance decreased from 100% to 60.5% within 2 h. Yang et al.^46^ proved that the physical adsorption of Hg^0^ could not get assistance from excessive temperature and could even cause a rerelease of the captured Hg^0^ from the surface of adsorbents, thereby weakening Hg^0^ removal. The above-mentioned experimental results indicated that the A_0.075_-Fe_0.4_-T850 adsorbent exhibited a wide temperature window of 60–150 °C, and 150 °C was considered as the optimal reaction temperature.

#### 3.1.3 Effect of Simulated Gas Components on Elemental Mercury Removal

On this basis, the Hg^0^ capture process is usually carried out in multicomponent flue gas. Thus, it is necessary to analyze the impact of flue gas components on Hg^0^ adsorption. The impact of two main acid gas components (SO_2_ and NO) on the removal of Hg^0^ by A_0.075_-Fe_0.4_-T850 adsorbent was analyzed at 150 °C. The impacts of SO_2_ and NO were analyzed when the oxygen volume fraction was 4%. Figure 1d shows the results. The Hg^0^ removal efficiency of A_0.075_-Fe_0.4_-T850 adsorbent in N_2_ atmosphere decreased from 86.9% to 55.3% within 2 h. Under N_2_ + O_2_ atmosphere, the removal efficiency of Hg^0^ reached 97.6% within 2 h. The removal efficiency of Hg^0^ in N_2_ + O_2_ + NO atmosphere was lower than that in N_2_ + O_2_ atmosphere, which indicated that NO could inhibit the Hg^0^ removal. It can be seen from Figure 1e that the Hg^0^ removal efficiency gradually decreased with the addition of NO from 200 to 800 ppm. In order to explore the inhibition mechanism of NO on the Hg^0^ removal performance of adsorbents, NO was intermittently injected into the N_2_ + O_2_ atmosphere during the evaluation of Hg^0^ removal efficiency of the adsorbent at 150 °C, and the change trend of the adsorbent Hg^0^ removal was recorded. It can be seen from Figure S1 that the mercury removal efficiency of the A_0.075_-Fe_0.4_ sample was maintained at 99.1% within 55 min in N_2_ + O_2_ atmosphere. When NO was introduced, the Hg^0^ removal efficiency of the A_0.075_-Fe_0.4_ sample significantly decreased. When NO was cut off, the Hg^0^ removal efficiency of the A_0.075_-Fe_0.4_ adsorbent increased. This could be attributed to competitive adsorption occurring between Hg^0^ and NO on the active site of the adsorbent.^47^

The impact of SO_2_ on Hg^0^ removal was difficult to explain because it may depend on the surface characteristics of adsorbent or flue gas components.^48^Figure 1f shows that the introduction of SO_2_ promoted the removal of Hg^0^. The removal efficiency of Hg^0^ was 100% within 2 h. When the SO_2_ concentration was 200, 400, 600, and 800 ppm, respectively, the Hg^0^ removal efficiency of the Fe/BC adsorbents remained 100% within 2 h. The existence of SO_2_ improved the removal efficiency of Hg^0^ of Fe/BC adsorbent. It can be seen from Figure 7 that there was a mercury release peak at 203 °C after the introduction of SO_2_, which was attributed to β-HgS.^49^Figure 6d shows the S 2p XPS spectra of fresh and used A_0.075_-Fe_0.4_-T850 samples. The peak at 169.4/169.3 eV was assigned to SO_4_^2–^, and the peak at 164.4 eV was assigned to active sulfur.^50,51^ It can be seen that the signal of sulfur was very weak in the fresh sample, and the fresh sample did not show the peak at 164.4 eV. However, the used adsorbent in SO_2_ atmosphere presented active sulfur at 164.4 eV, which was beneficial for mercury removal.^50,51^ Therefore, the effects of SO_2_ on the removal of Hg^0^ over Fe/BC adsorbents can be concluded: SO_2_ was adsorbed on the surface of Fe/BC adsorbents, and could dissociate active sulfur sites and oxygen sites on the surface of Fe_2_O_3_.^52^ Finally, the dissociated active sulfur on the surface of Fe/BC adsorbents could react with Hg to form β-HgS.^49^

#### 3.1.4 Comparison of the Fe/BC Adsorbents with Other ACs/Modified ACs

The characteristics of some magnetic biomass-based activated carbon including tea, cotton straw, rice straw, maize straw, and pinewood sawdust are shown in Table S1. These data are used for comparison with this study. Compared with other magnetic biomass-based activated carbon, Fe/BC adsorbent showed good Hg^0^ removal performance and magnetic performance. The regenerated Fe/BC adsorbent remained as a good superparamagnetic structure after the four cycles and its saturation magnetization remained at 16.7 emu g^–1^, which promoted the recovery of the adsorbent after use. The preparation methods reported in the literature were relatively complicated. In this work, the magnetic Fe-modified porous carbon was prepared by the one-step method and the adsorbents prepared have high Hg^0^ adsorption performance and magnetic performance.

### 3.2 Characteristics of Iron-Modified Biomass Carbon Adsorbent

#### 3.2.1 Analysis of Pore Property

**Figure 2:**
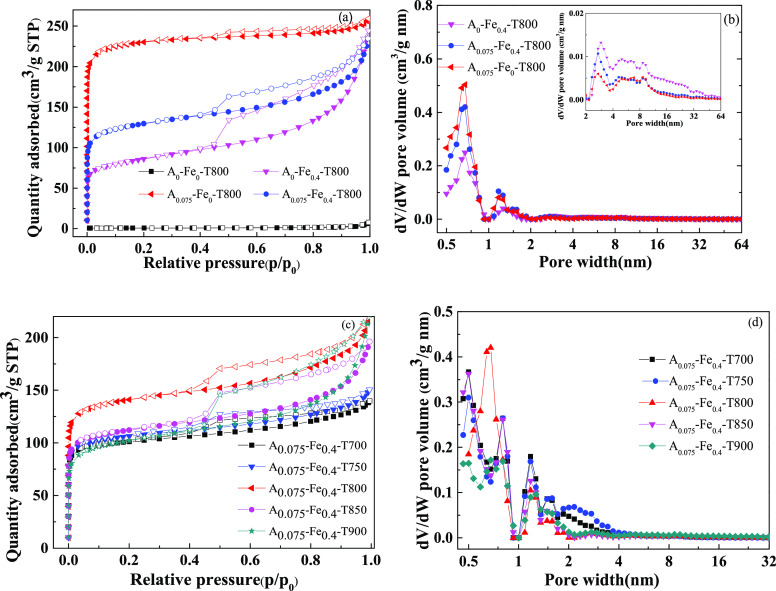
(a) N_2_ adsorption isotherms and (b) pore size distributions of different adsorbents; (c) N_2_ adsorption–desorption isotherms and (d) pore size distributions of Fe/BC under different activation temperatures.

In order to analyze the texture characteristics of Fe/BC adsorbent, the N_2_ adsorption desorption isotherm of the adsorbent was measured. Figure 2 shows the results. Table 2 lists the BET surface area (*S*_BET_), average pore diameter (*D*_P_), and pore volume (*V*_P_). Parts a and c of Figure 2 demonstrated that N_2_ adsorption isotherms of Fe/BC rapidly increased in the low-pressure area and showed an upward trend in the higher relative pressure range. This was attributed to micropore filling. With the distribution of pore size, it was found that many micropores were in the pore width less than 2.0 nm in Fe/BC adsorbents. It can be seen from Table 2 that the specific surface area after chemical activation was much larger than that of the inactivated one (sample A_0_-Fe_0_). The specific surface area of sample A_0_-Fe_0_ was only 3.95 m^2^ g^–1^, which might lead to its low removal performance of Hg^0^ (about 0.4%). Addition of only K_2_C_2_O_4_ mainly resulted in the generation of micropores, with a large *S*_BET_ of 913.08 m^2^ g^–1^. In contrast, when only Fe(NO_3_)_3_ was added, the *S*_BET_ of pepper straw was 315.43 m^2^ g^–1^, and 4.8 nm was the *D*_P_. However, when the two coexisted, the *S*_BET_ of pepper straw was 543.15 m^2^ g^–1^, and the D_P_ was 2.5 nm, which facilitated the removal of Hg^0^ from the flue gas.^53^ It may be attributed to the fact that when Fe(NO_3_)_3_ was added during the biomass activation process it decomposed into Fe_2_O_3_, which affected the performance of Hg^0^ removal of Fe/BC adsorbent. The *S*_BET_ of Fe/BC first increased and then decreased, and the *V*_P_ and *D*_P_ gradually increased when the activation temperature gradually increased from 750 to 900 °C. Figure 1b illustrates that A_0.075_-Fe_0.4_-T850 showed greater capability of Hg^0^ capture than the others. However, the *S*_BET_ of the A_0.075_-Fe_0.4_-T850 adsorbent was less than that of A_0.075_-Fe_0.4_-T800, indicating that physical adsorption existed in the Hg^0^ removal process.

**Table 2:** Pore Structure Characteristics of Fe/BC Adsorbents

sample name	surface area (m^2^ g^–1^)	*V*_P_ (cm^3^ g^–1^)	*D*_P_ (nm)
A_0_-Fe_0_-T800	3.95	0.02	16.71
A_0.075_-Fe_0_-T800	913.08	0.40	1.76
A_0_-Fe_0.4_-T800	315.43	0.37	4.80
A_0.075_-Fe_0.4_-T800	543.15	0.34	2.53
A_0.075_-Fe_0.4_-T700	394.35	0.22	2.19
A_0.075_-Fe_0.4_-T750	413.01	0.23	2.26
A_0.075_-Fe_0.4_-T850	428.72	0.30	2.83
A_0.075_-Fe_0.4_-T900	379.09	0.34	3.62

#### 3.2.2 Analysis of X-ray Diffraction

**Figure 3:**
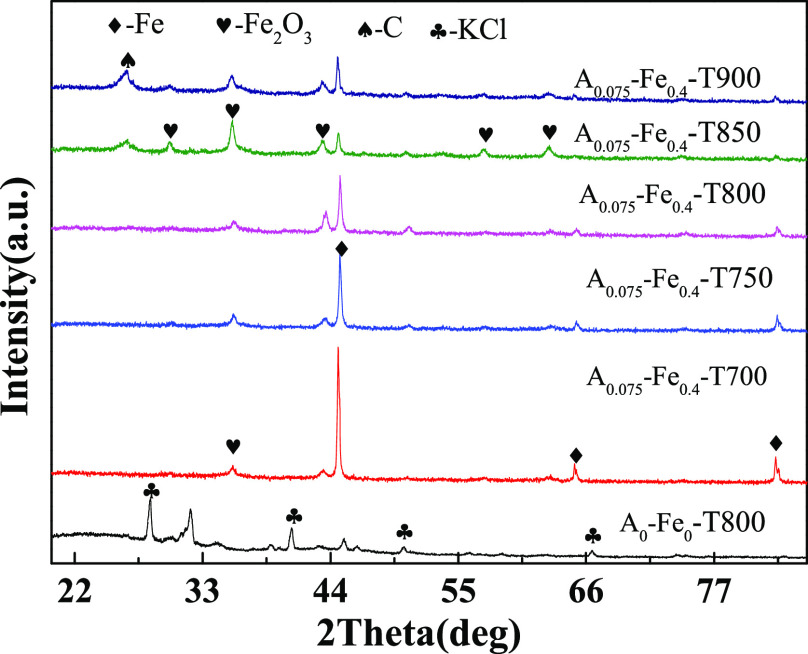
XRD patterns of raw pepper straw and Fe/BC under different activation temperatures.

Figure 3 shows that powder XRD patterns of A_0_-Fe_0_-T800 adsorbent and A_0.075_-Fe_0.4_ adsorbents at different activation temperatures were obtained. The diffraction peaks of A_0_-Fe_0_-T800 sorbent at 28.4°, 40.6°, 50.3°, and 66.5° were attributed to KCl. It is relevant to the high ash content of raw pepper straw. Figure 3 showed the presence of obvious characteristic diffraction peaks of five types of Fe/BC samples at 44.7°, and it corresponded to elemental Fe.^42^ The source of elemental Fe could be explained as follows: K_2_C_2_O_4_ decomposed into K_2_CO_3_ at 600 °C, and the generated K_2_CO_3_ further corroded carbon and then released CO. In the process of carbonization, Fe(NO_3_)_3_ was resolved to Fe_2_O_3_, and the Fe_2_O_3_ was reduced by CO and gradually was transformed into Fe.^42^ The diffraction peaks of A_0.075_-Fe_0.4_-T850 adsorbent at 30.5°, 35.9°, 43.4°, 57.5°, and 63.2° were attributed to Fe_2_O_3_. The distinctive peak intensity of Fe decreased little by little as the temperature gradually increased from 750 to 900 °C. A peak temperature of 850 °C was found for graphite carbon. It can also be seen from Figure S2 that the full width at half maxima of G peak of adsorbent decreased and that of D peak increased at 850 °C, compared with other temperatures. That was indicated the graphitization degree of adsorbent increased. This result could explain why the *S*_BET_ of sample A_0.075_-Fe_0.4_-T850 was lower than that of sample A_0.075_-Fe_0.4_-T800,^54^ which was consistent with the result of XRD.

#### 3.2.3 Magnetization Analysis

**Figure 4:**
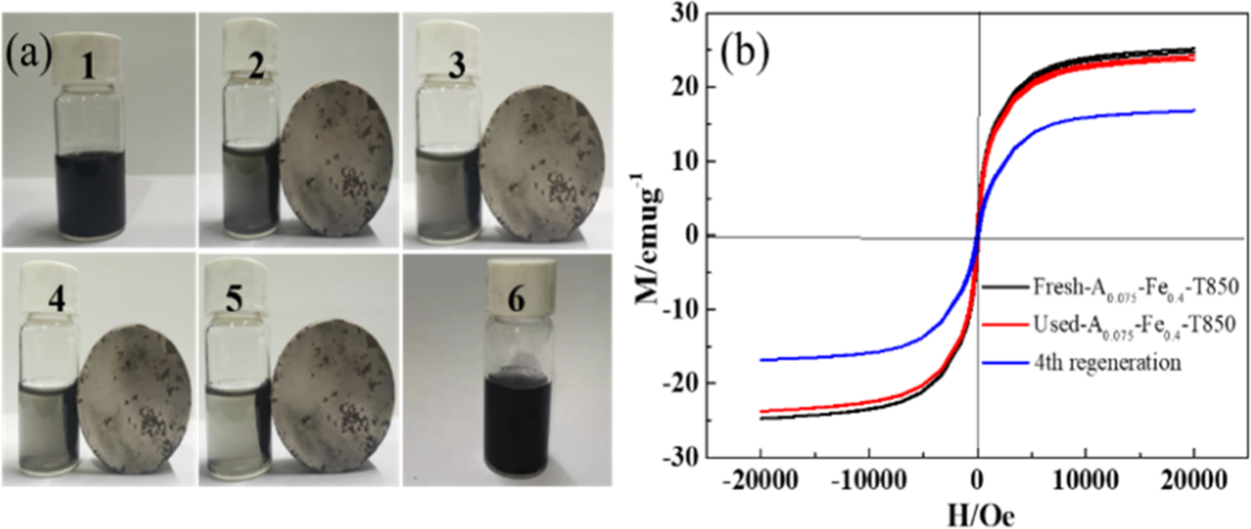
Magnetization characteristics: experimental photographs of A_0.075_-Fe_0.4_-T850 sample under an external magnetic field (a); A_0.075_-Fe_0.4_-T850, used A_0.075_-Fe_0.4_-T850, and fourth-regeneration adsorbents (b).

Figure 4a shows photos of the magnetic response experiment performed on the aqueous solution of adsorbent under the action of an external magnetic field. Here, no. 1 is the photograph of adsorbent A_0.075_-Fe_0.4_-T850 in aqueous solution for 2 h. The A_0.075_-Fe_0.4_-T850 sample was basically uniformly dispersed in aqueous solution. Figure 4a nos. 2–5 are photographs of the magnetic response of adsorbent A_0.075_-Fe_0.4_-T850 in the external magnetic field at different times (the time interval is 10 s). Figure 4a no. 6 is the photograph after removing the external magnetic field, indicating that the sample could be redispersed without the external magnetic field. It was found that with the action of the magnetic field, the adsorbent continuously gathered toward the side of the magnetic area, indicating that the A_0.075_-Fe_0.4_-T850 adsorbent exhibited a significant magnetic response characteristic, which facilitated the recovery of the adsorbent after use.

For further studying the magnetic performance of A_0.075_-Fe_0.4_-T850 sample, VSM was used to obtain and evaluate the magnetization curve of the sample. Figure 4b demonstrated that the Fe/BC adsorbent showed a coercivity in the smallest degree and a magnetization hysteresis which could be ignored, thus indicating that it could be regarded as a superparamagnetic material. The adsorbent Fresh-A_0.075_-Fe_0.4_-T850 showed good magnetization and saturation magnetization of 25 emu g^–1^. There was no obvious variation of the magnetism between Used-A_0.075_-Fe_0.4_-T850 sample and Fresh-A_0.075_-Fe_0.4_-T850 sample. After four regenerations, the magnetism of A_0.075_-Fe_0.4_-T850 adsorbent was found to weaken. The magnetization property could prevent the sample from being permanently magnetized, which was conducive to the redispersion of sample without the external magnetic field.^35^ This result indicated that when an external magnetic field was introduced, it was possible to recover the used A_0.075_-Fe_0.4_-T850 adsorbent from fly ash.

#### 3.2.4 Scanning Electron Microscopy Analysis

**Figure 5:**
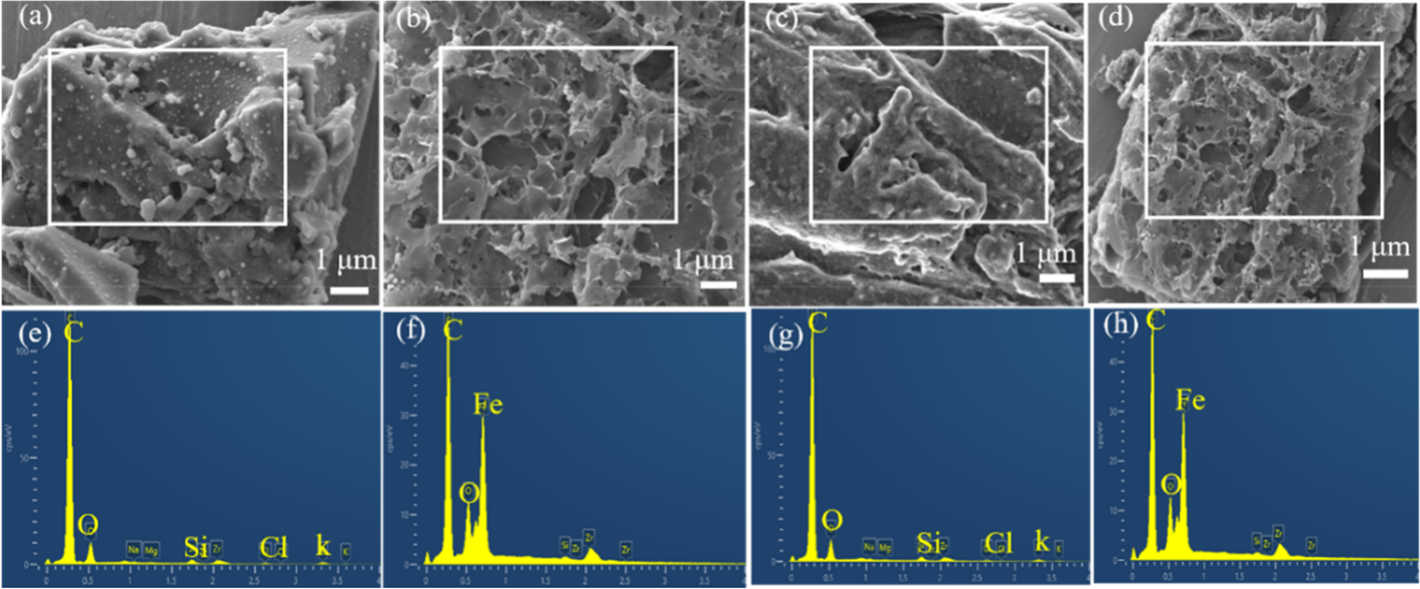
SEM images of (a) A_0_-Fe_0_-T800 and (b) A_0.075_-Fe_0.4_-T800; (c) A_0_-Fe_0_-T850; and (d) A_0.075_-Fe_0.4_-T850 and EDS spectra of (e) A_0_-Fe_0_-T800 and (f) A_0.075_-Fe_0.4_-T800; (g) A_0_-Fe_0_-T850; and (h) A_0.075_-Fe_0.4_-T850.

Figure 5 present the SEM images and EDS spectra of A_0_-Fe_0_-T800, A_0.075_-Fe_0.4_-T800, A_0_-Fe_0_-T850, and A_0.075_-Fe_0.4_-T850. Compared to A_0_-Fe_0_ sample, the surface of the activated sample was loose and porous, which was beneficial for Hg^0^ removal ability of adsorbents.^53^ It can be seen from the EDS spectra in Figure 5f,h that the samples activated by Fe(NO_3_)_3_ mainly contained the elements of C, O, and Fe. In contrast, the composition of the samples without Fe(NO_3_)_3_ treatment was C, K, Cl, Si, and O.

### 3.3 Mechanism Discussion

#### 3.3.1 Analysis of X-ray Photoelectron Spectroscopy

**Figure 6:**
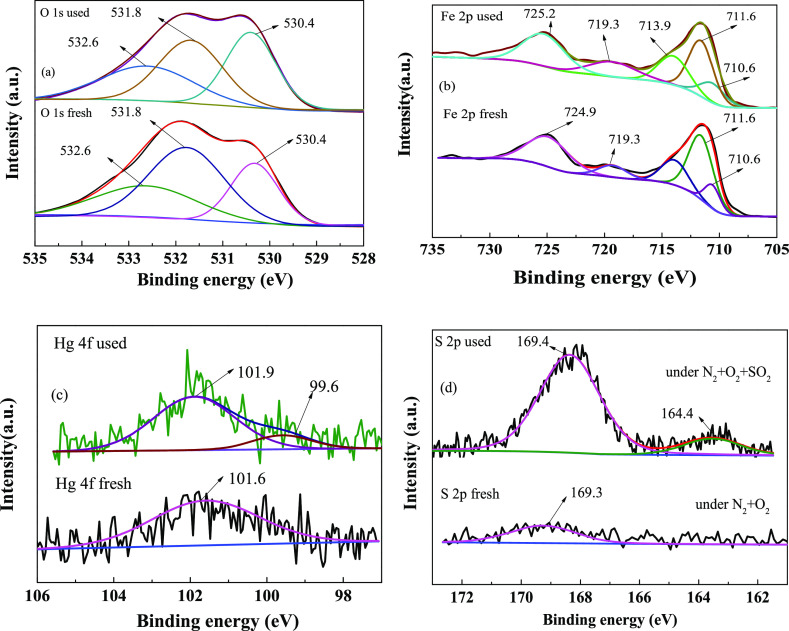
XPS analysis of fresh and used Fe/BC adsorbents: (a) O 1s, (b) Fe 2p, (c) Hg 4f, and (d) S 2p.

To clarify the removal mechanism of Hg^0^ of the Fe/BC adsorbent, the analysis of XPS was used to decide the valence states of the surface elements of the fresh and used A_0.075_-Fe_0.4_-T850 adsorbent. Figure 6 shows the XPS spectra of O 1s, Fe 2p, Hg 4f, and S 2p. The O 1s XPS spectrum of fresh and used A_0.075_-Fe_0.4_-T850 adsorbent is presented in Figure 6a. It shows three peaks for fresh and used adsorbents. Approximately 530.4 eV was accessed to the characteristic peak of lattice oxygen (O_α_). The peak at 531.8/531.7 eV was contributed to chemically adsorbed oxygen (O_β_), and the peak at 532.6 eV was contributed to molecular water (O_γ_). Yang et al.^46^ suggested that the existence of O_α_ may be result from the existence of metal oxides, and the existence of O_β_ was relevant to the charge imbalance, vacancies and chemical bonds produced by metal oxides. Table 3 make a summary after the removal experiment of Hg^0^. The proportion of O_α_ increased from 28.6% to 30.5%, while the proportion of O_β_ reduced from 46.9% to 36.1%. These data indicated that O_β_ plays important role for Hg^0^ removal.

**Table 3:** Contents of Fe and O Species on Fe/BC Adsorbent Surface Based on XPS Analysis

			relative content (%)	
species		position (eV)	fresh sample	used sample
	O_(γ)_	532.6	28.9	30.2
O	O_(α)_	530.4	24.5	33.4
	O_(β)_	531.8	46.9	36.1
Fe	Fe^2+^	710.6	14.2	17.7
	Fe^3+^ (octahedral)	711.6	60.2	50.2
	Fe^3+^ (tetrahedral)	713.9/714.1	25.5	32.1
	Fe^3+^/Fe^2+^		6.0	4.6

Figure 6b shows the Fe 2p XPS spectra of fresh and used A_0.075_-Fe_0.4_-T850 samples. The three subpeaks at 710.6, 711.6, and 714.1/713.9 eV (belonging to Fe 2p3/2) were correspond to Fe^2+^, Fe^3+^ (octahedron), and Fe^3+^ (tetrahedron), respectively.^46^ The ratio of Fe^3+^/Fe^2+^ concentration dropped from 6.0% to 4.6% (Table 3) after removal of Hg^0^. The results showed that Fe^3+^ was reduced to Fe^2+^ during the Hg^0^ capture process.

Figure 6c shows the results. It was the Hg 4f XPS spectrum of this fresh and used A_0.075_-Fe_0.4_-T850 sample. The peak centered at 99.6 eV was designated as Hg^0^, and the peak centered at 101.9 eV was designated HgO.^55^ Thus, it could be inferred that O_β_ and O_α_ were important in the Hg^0^ removal by the A_0.075_-Fe_0.4_-T_850_ adsorbent.

#### 3.3.2 Analysis of Hg-Temperature-Programmed Desorption

**Figure 7:**
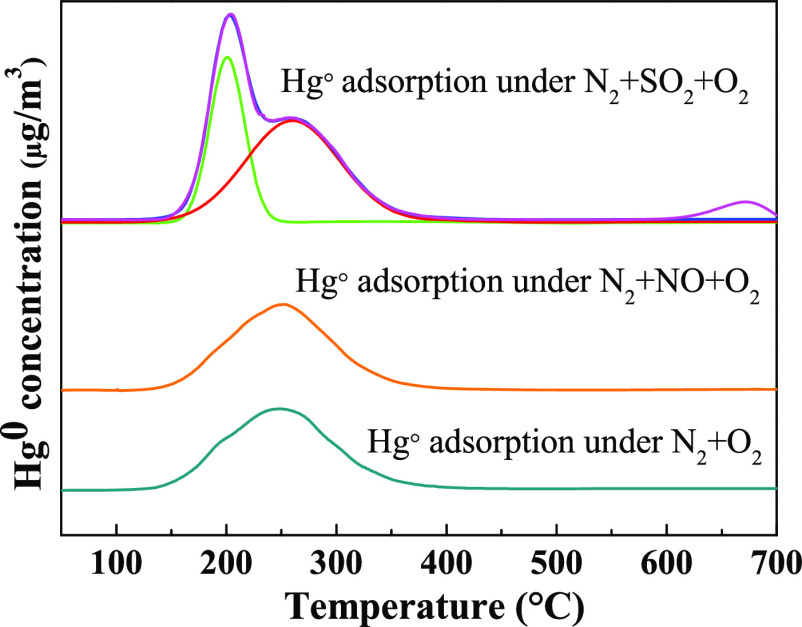
Hg-TPD curves of used A_0.075_-Fe_0.4_-T850 adsorbent.

To identify the types of Hg adsorbed on the A_0.075_-Fe_0.4_-T850 adsorbent, an Hg-TPD experiment was carried out. It is worth mentioning that as a simple and available method Hg-TPD can clarify mercury species adsorbed on solid adsorbents. Figure 7 suggested the Hg-TPD curve of A_0.075_-Fe_0.4_-T850 adsorbent after capturing Hg^0^ in various atmospheres at 150 °C. It was found that under a N_2_ + O_2_ atmosphere and temperature of 247 °C there was a desorption peak that contributed to HgO.^56^ After the introduction of SO_2_, there was a mercury release peak at 203 °C, which was attributed to β-HgS.^49^ When NO was introduced into the simulated flue gas (N_2_ + O_2_), only one peak appeared at 250 °C, which was assigned to HgO.

#### 3.3.3 Elemental Mercury Adsorption Mechanism over Iron-Modified Biomass Carbon Adsorbent

On the basis of the removal efficiency of Hg^0^ and characteristic results, the mechanism of Hg^0^ removal over Fe/BC adsorbents can be concluded in two ways in N_2_ + O_2_: First, gaseous Hg^0^ was adsorbed on the surface of the sample to form Hg^0^(ad) (eq 2). In addition, the generation process of lattice oxygen could be described by eq 3. Then, lattice oxygen reacted with Hg^0^(ad) adsorbed on the surface of the sample to generate HgO by several reactions (eq 4). The second was that O_2(g)_ was adsorbed on the surface of the adsorbent to form O_2(ad)_, and the adsorbed Hg^0^ could be oxidized to form HgO by O_2(ad)_. Finally, FeO was reoxidized to Fe_2_O_3_ by gas-phase O_2_. The consumed chemisorbed oxygen and lattice oxygen could be replenished by the O_2_ in the gas phase, which can form an oxygen cycle during the Hg^0^ capture process.^35^ The reaction can be described by eqs 5–7.
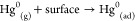
2
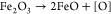
3
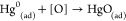
4
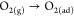
5
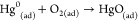
6
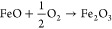
7

### 3.4 Regeneration Performance of Iron-Modified Biomass Carbon Adsorbents

**Figure 8:**
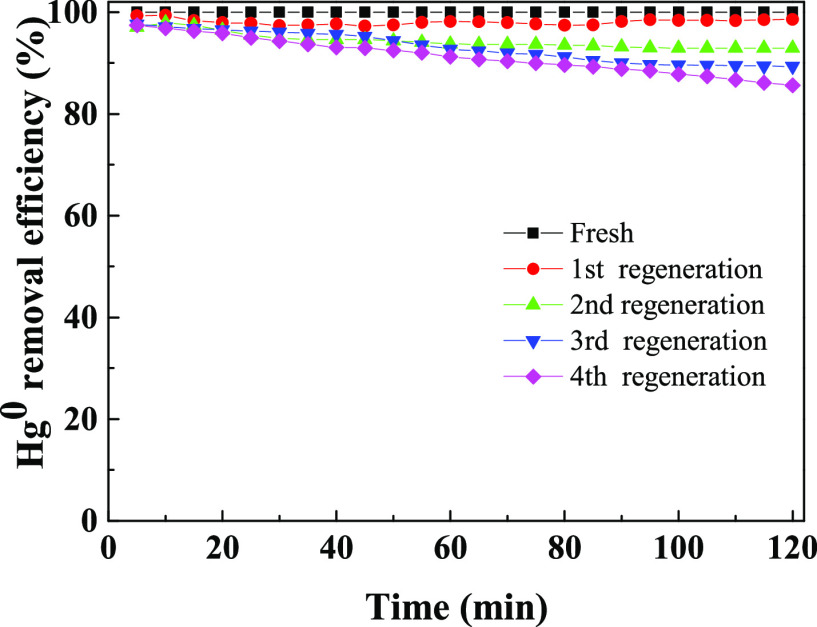
Performance of Hg^0^ removal over Fe/BC adsorbents after regeneration. Experimental conditions: Hg^0^ inlet concentration 40 ± 2 μg m^–3^, 4 vol % O_2_, 200 ppm of SO_2_ (when used), 400 ppm of NO (when used), 5 vol % H_2_O (when used) *T* = 150 °C.

The regenerability and reusability of used adsorbents are very important in practical industrial applications. Thus, in this study, the recycle tests were conducted on used A_0.075_-Fe_0.4_-T850 adsorbent. The used A_0.075_-Fe_0.4_-T850 adsorbent was first heated to 500 °C in N_2_ to decompose HgO on the sample. When the Hg^0^ concentration at the outlet the online Hg analyzer was lower than 0.3 μg m^–3^, the removal performance of Hg^0^ of the regenerated A_0.075_-Fe_0.4_-T850 adsorbent could be accessed in N_2_–O_2_–SO_2_–NO–H_2_O–Hg atmosphere at 150 °C. This was considered as a one-cycle process, and the same cycle process was repeated for four times. Figure 8 shows the results. After the first cycle, the removal performance of Hg^0^ of regenerated adsorbent remained above 98.6% within 2 h, which was close to that of the fresh sample. The removal performance of Hg^0^ of regenerated adsorbent was reduced; however, it was above 92.9%. After four cycles, the removal performance of Hg^0^ of the regenerated sample was still more than 85%. These results showed that the A_0.075_-Fe_0.4_-T850 adsorbent was of great regeneration efficiency, which improved the utilization rate and reduced the cost of the removal of Hg^0^.

The magnetic properties of the adsorbent are discussed in section 3.2.3. Herein, the magnetism of the regenerated adsorbent after four cycles was detected. Figure 4b illustrates that the regenerated adsorbent remains as a good superparamagnetic structure after four cycles and its saturation magnetization is lower than that of the fresh sample (25.0 emu g^–1^) but still remains at 16.7 emu g^–1^. Wang et al.^57^ reported that the saturation magnetization value of magnetic carbon composite derived from pinewood sawdust was 15.6 emu g^–1^, indicating that the separation of magnetic carbon composite was facile, and almost all adsorbent could be completely recollected using a magnet. Zou et al.^58^ reported that the improved Fe–Ti spinel remained as a great superparamagnetic structure. The saturation magnetization of it has decreased from 24.6 to 11.8 emu g^–1^ after the four cycles of Hg^0^ capture/recovery. These results indicated that the Fe/BC sample exhibited good magnetization performance, which received assistance from an external magnetic field to separate from fly ash.

## 4 Conclusion

In this study, Fe/BC magnetic adsorbent derived from pepper straw was prepared by a one-step method with K_2_C_2_O_4_ and Fe(NO_3_)_3_ as activator and catalyst precursor, respectively. Its Hg^0^ removal efficiency was studied in the fixed-bed system. When the activation temperature was 850 °C, the removal performance of Hg^0^ of iron-modified biomass carbon (Fe/BC) adsorbent was excellent, above 97.6% at 150 °C. The Fe/BC showed a wide temperature range for capturing Hg^0^. The addition of SO_2_ into simulated flue gas (N_2_ + O_2_) resulted in efficient improvement in the removal performance of Hg^0^. The Hg^0^ removal efficiency of Fe/BC adsorbent increased to 100% at 150 °C under N_2_ + O_2_ + SO_2_. Nevertheless, addition of NO resulted in significant inhibition of Hg^0^ capture. The Hg^0^ removal efficiency of the magnetic Fe/BC adsorbent increased first and then decreased with the increase of the reaction temperature. Regarding the Hg^0^ adsorption mechanism, the lattice oxygen in Fe_2_O_3_, and chemically adsorbed oxygen as the active site for Hg^0^ removal, the Hg species on the used Fe/BC adsorbents was HgO. The results of experiments suggested that after four capture–regeneration cycles Fe/BC adsorbent showed a good regeneration and magnetization performance; these results indicated that pepper straw can be used as a potential raw material for the preparation of biomass carbon adsorbents.

## Supporting Information Available

Effect of NO intermittent supply for Hg^0^ removal performance of Fe/BC adsorbent, Raman spectra of Fe/BC adsorbent, the comparison of Hg^0^ adsorption capacity between Fe/BC and other biomass AC (PDF)
