# Supplementary material for: Biomass Carbon Magnetic Adsorbent Constructed by One-Step Activation Method for the Removal of Hg0 in Flue Gas
Source: ACS Omega. 2022 Mar 10;7(11):9244–53. doi: 10.1021/acsomega.1c05857 (PMC8945062; doi:10.1021/acsomega.1c05857)
Supplement: Supplementary file 1 — ao1c05857_si_001.pdf [file ao1c05857_si_001.pdf]

Supporting Information for

Biomass Carbon Magnetic Adsorbent Constructed by One-Step Activation Method for the Removal of Hg^0 in Flue Gas

Yu Cui[a], Qihuang Huo[b], Huijun Chen[b], Shuai Chen[c], Sheng Wang[d], Jiancheng Wang[b], Liping Chang[b], Lina Han[a,*], Wei Xie[e,**]

[a]*College of Materials Science and Engineering, Taiyuan University of Technology, Taiyuan 030024, China*

[b]*State Key Laboratory of Clean and Efficient Coal Utilization, Taiyuan University of Technology, Taiyuan 030024, China*

[c]*Analytical Instrumentation Center, Institute of Coal Chemistry, Chinese Academy of Sciences, Taiyuan, China.*

[d]*Dalian National Laboratory for Clean Energy, Dalian Institute of Chemical Physics, Chinese Academy of science, Dalian 116023, China*

[e] *Chemical Engineering, University of Newcastle, Callaghan NSW 2308, Australia*

*Corresponding Author: Lina Han, E-mail: hanlina@tyut.edu.cn (L. N. Han)

** Corresponding Author: Wei Xie

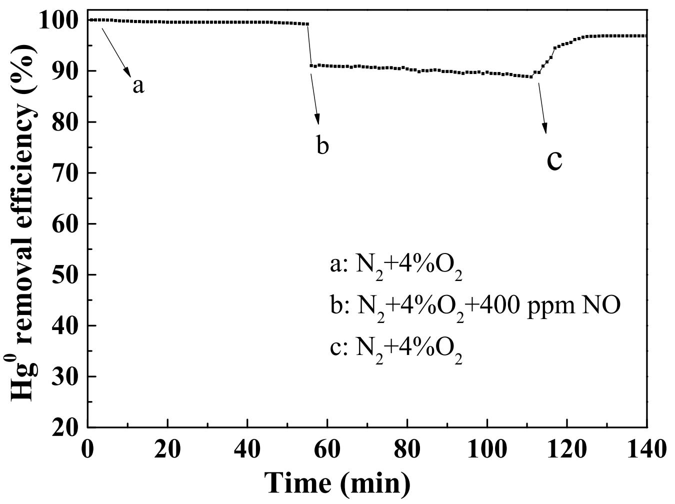

Figure. S1. Effect of NO intermittent supply for Hg^0 removal performance of Fe/BC adsorbent. Reaction conditions: T=150 °C, in the atmosphere: 40 ± 2 μg·m^{-3} Hg^0, 4vol% O_2, 400 ppm NO (when in use), 600 mL·min^{-1} carrier N_2.

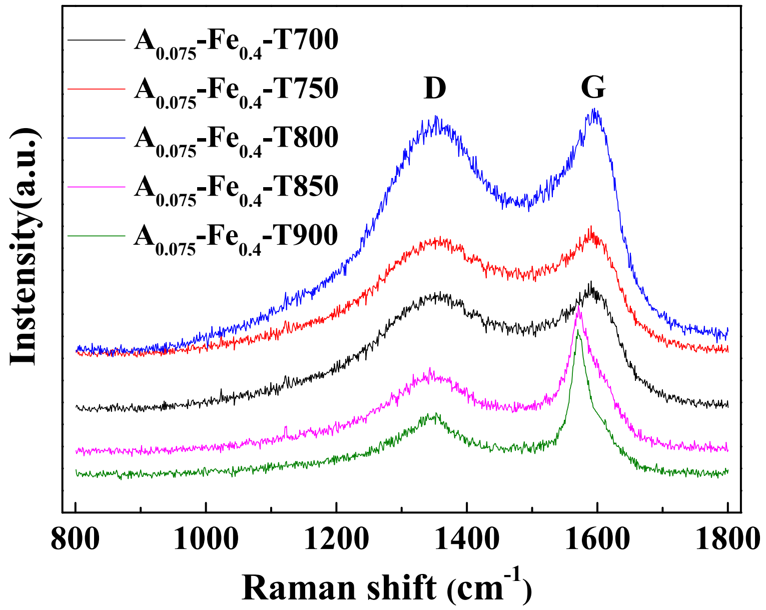

Figure. S2 Raman spectra of Fe/BC adsorbent.

Table S1 The comparison of Hg^0 adsorption capacity between Fe/BC and other biomass AC.

original material	Preparation method	Hg^{RE}(%)	Magnetization (emu g^{-1})
Tea[40]	one-step synthesis	96	15
Cotton straw[39]	pyrolysis, microwave activation, chemical modification	87.1	0.4
Rice straw[35]	pyrolysis, microwave and water steam activation, chemical modification	86.6	0.5
Maize straw[37]	pyrolysis, microwave action, chemical modification	95.6	0.4
Pinewood Sawdust[57]	hydrothermal, calcination,	98	15.6
**Pepper straw(in this manuscript)**	**one-step method**	**97.6**	**25**
